# Tuning the Orchestra: HCMV vs. Innate Immunity

**DOI:** 10.3389/fmicb.2020.00661

**Published:** 2020-04-15

**Authors:** Valentina Dell’Oste, Matteo Biolatti, Ganna Galitska, Gloria Griffante, Francesca Gugliesi, Selina Pasquero, Alessandra Zingoni, Cristina Cerboni, Marco De Andrea

**Affiliations:** ^1^Laboratory of Pathogenesis of Viral Infections, Department of Public Health and Pediatric Sciences, University of Turin, Turin, Italy; ^2^Department of Molecular Immunology and Immunopathology, “Sapienza” University of Rome, Rome, Italy; ^3^Center for Translational Research on Autoimmune and Allergic Disease – CAAD, University of Piemonte Orientale, Novara, Italy

**Keywords:** human cytomegalovirus, innate immunity, interferon system, apoptosis, restriction factors, NK cells, antigen presenting cell (APC)

## Abstract

Understanding how the innate immune system keeps human cytomegalovirus (HCMV) in check has recently become a critical issue in light of the global clinical burden of HCMV infection in newborns and immunodeficient patients. Innate immunity constitutes the first line of host defense against HCMV as it involves a complex array of cooperating effectors – e.g., inflammatory cytokines, type I interferon (IFN-I), natural killer (NK) cells, professional antigen-presenting cells (APCs) and phagocytes – all capable of disrupting HCMV replication. These factors are known to trigger a highly efficient adaptive immune response, where cellular restriction factors (RFs) play a major gatekeeping role. Unlike other innate immunity components, RFs are constitutively expressed in many cell types, ready to act before pathogen exposure. Nonetheless, the existence of a positive regulatory feedback loop between RFs and IFNs is clear evidence of an intimate cooperation between intrinsic and innate immunity. In the course of virus-host coevolution, HCMV has, however, learned how to manipulate the functions of multiple cellular players of the host innate immune response to achieve latency and persistence. Thus, HCMV acts like an orchestra conductor able to piece together and rearrange parts of a musical score (i.e., innate immunity) to obtain the best live performance (i.e., viral fitness). It is therefore unquestionable that innovative therapeutic solutions able to prevent HCMV immune evasion in congenitally infected infants and immunocompromised individuals are urgently needed. Here, we provide an up-to-date review of the mechanisms regulating the interplay between HCMV and innate immunity, focusing on the various strategies of immune escape evolved by this virus to gain a fitness advantage.

## Introduction

The innate immune response is a fundamental defense mechanism, shielding the host from constant attacks of invading pathogens of different origin, whether they are bacterial, fungal, transposon or viral (Akira et al., 2006; Yan and Chen, 2012). Thus, for a virus, successful invasion and efficient subversion of the host immediate immune response are critical steps to achieve productive infection.

Some viruses, such as herpesviruses, have succeeded in establishing lifelong persistence in humans by evading immune surveillance (Stempel et al., 2019). For example, human cytomegalovirus (HCMV), a notorious opportunistic pantropic betaherpesvirus with a worldwide seroprevalence of 50 to > 90% in adults (Cannon et al., 2010), has the remarkable ability to manipulate and evade immune detection, literally transforming the host cellular environment into an ideal niche in which to thrive (Griffiths et al., 2015). This is achieved through sophisticated manipulations of cellular gene expression or elegant evasion strategies evolved by the virus during its long lasting co-evolution with the host (Wang et al., 2007; Loewendorf and Benedict, 2010; Rossini et al., 2012).

Even though HCMV infection is asymptomatic in immunocompetent individuals, it may lead to several life-threatening conditions in immunosuppressed subjects, such as organ and stem cell transplant recipients or AIDS patients. Furthermore, it can cause severe morbidity in congenitally infected children and elderly people (Cannon et al., 2010; Manicklal et al., 2013; Tu and Rao, 2016; Britt, 2018). Additionally, spontaneous reactivation of latent endogenous virus and/or superinfection with multiple viral strains can contribute to the overall burden and individual disease severity, as neither a vaccine nor an effective cure is currently available (Schleiss et al., 2017).

Although several viral polymerase inhibitors acting upon lytic replication (e.g., ganciclovir, cidofovir, and foscarnet) are widely used to treat HCMV infections, they are characterized by high hematopoietic toxicity and poor bioavailability, which prevents their use in pregnant women and congenitally infected newborns (Britt and Prichard, 2018). In addition, targeting latent HCMV remains an unsolved issue in patient clinical management. To make matters worse, the number of drug-resistant HCMV mutants has increased dramatically over the last decade (Piret and Boivin, 2019).

The outcome and severity of HCMV infection depends predominantly on initial virus-host interactions, occurring early upon infection, when intrinsic innate immunity comes into play to fight off the virus. As a frontline defense and earliest reaction measure, innate immunity avail itself of a complex array of effector cells and soluble factors, including pro-inflammatory cytokines and type I interferon (IFN-I), natural killer (NK) cells, professional antigen-presenting cells (APCs) and phagocytes, all operating in a fine-tuned and balanced manner (Luecke and Paludan, 2015; Patel et al., 2018).

Intrinsic cellular restriction factors (RFs) are constitutively expressed and play physiological roles in uninfected cells by cooperating with innate immune effectors, as some of them appear to be IFN-inducible, thus contributing to early host defense (Bieniasz, 2004; Duggal and Emerman, 2012).

Finally, triggered cell suicide processes (i.e., apoptosis and pyroptosis), resulting in death and removal of HCMV-infected cells, can also have a major impact on viral infection progression (Brune and Andoniou, 2017).

Ultimately, the orchestra formed by these innate immune components fine-tunes a highly efficient adaptive immune response that keeps HCMV infection at bay. However, HCMV often becomes the conductor of this orchestra, and as such it can manipulate to its liking all the various components of the immune response to make the cellular environment more permissible to viral replication and survival, thereby achieving persistence, latency and ultimately seroprevalence.

HCMV has an extremely large genome, and its enhanced encoding capacity allows for generating multiple viral proteins, involved in modulation and subversion of multiple signaling pathways (Stern-Ginossar et al., 2012; Brune and Andoniou, 2017). The exact mechanisms of action and role of this large number of viral proteins have not been yet completely elucidated, although many of them are probably involved in immune evasion.

In this regard, the fact that HCMV has developed a number of ingenious strategies directed against NK cells and APCs underscores the overall importance of these cells in innate immunity. For example, NK cells can release cytotoxic granules triggered by natural or antibody-dependent cytotoxicity (ADCC) or produce cytokines upon engagement of activating and inhibitory NK cell receptors. Even though NK cells are the major cytotoxic arm of innate immunity, their contribution in shaping T cell-mediated immune responses and generating memory cells is now well established (Netea et al., 2016; Nikzad et al., 2019). Since NK cells are efficient eliminators of HCMV-infected cells, it is not surprising that HCMV has devised multiple strategies to evade recognition by these cells (Babić et al., 2011; Goodier et al., 2018; Zingoni et al., 2018). Likewise, APCs from the myeloid and epithelial compartments [i.e., monocytes, macrophages, and dendritic cells (DCs)], are well-known targets of HCMV, serving as vehicles upon infection to facilitate viral dissemination (Jackson and Sparer, 2018). In particular, HCMV is able to interfere with MHC class I (MHC-I) and II (MHC-II) antigen presentation, thereby subverting the immunological functions of APCs.

This review provides an in-depth description of the complex interplay between the host innate immune responses and HCMV, highlighting multiple viral feedback mechanisms that modulate and counteract the various arms of innate immunity.

## The IFN System and HCMV: A Stormy Relationship

Upon HCMV sensing, intracellular pattern recognition receptors (PRRs) trigger downstream signaling events leading to the production of type I IFN and release of inflammatory cytokines. Type I IFNs (IFN-I) are a group of cytokines comprising IFN-α, IFN-β, IFN-ε, IFN-κ, IFN-ω, IFN-δ, IFN-ζ, and IFN-τ (Mesev et al., 2019).

IFN-I signaling pathways have long been considered key limiting factors of HCMV infection and replication. Despite their complexity, these defense mechanisms occur early after pathogen entry into the host and, in most cases, they can eradicate the pathogen before it can overwhelm the host immune defenses (Goodwin et al., 2018).

**FIGURE 1:**
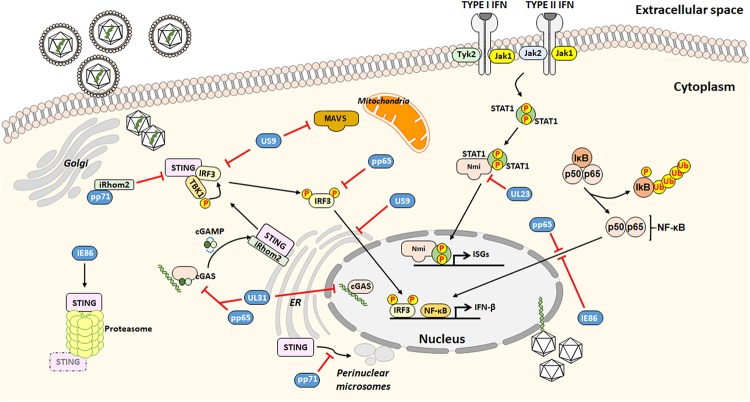
Outline of the HCMV strategies to evade from the interferon (IFN)-associated antiviral activity.

Cellular sensors capable of detecting HCMV include toll-like receptor 2 (TLR2) and CD14 receptors, both able to interact with HCMV envelope glycoproteins (Compton et al., 2003), most of DNA sensors and the newly described group of PRRs, able to stimulate transcription of IFN-I *via* the key adaptor protein stimulator of interferon genes (STING). In particular, the DNA sensor cyclic guanosine monophosphate (GMP)–adenosine monophosphate (AMP) synthase (cGAS)/STING axis is crucial for activating the IFN-I signaling (Diner et al., 2016; Paijo et al., 2016; Jønsson et al., 2017; Biolatti et al., 2018b). On the other hand, HCMV has evolved a wide range of proteins with which to manipulate and counteract the host IFN response (Biolatti et al., 2018c; Goodwin et al., 2018; Marques et al., 2018; Stempel et al., 2019). This complex and intertwined relationship between HCMV and IFN has been addressed by a number of studies discussed below and schematically represented in Figure 1.

The HCMV tegument protein pp65 –also identified as pUL83, encoded by *UL83* – best exemplifies the multifaceted interplay between viral and host proteins (Biolatti et al., 2018a). Specifically, pp65 has been shown to modulate nuclear factor-κB (NF-κB) and interferon regulatory factors 3 (IRF3) activities, which cooperate to induce transcription of several cytokines such as IFN-β, which then counteracts HCMV infection (Iwanaszko and Kimmel, 2015).

The recent finding that IκB kinases, the main regulators of NF-κB pathway, exerts antiviral activity (Goodwin and Munger, 2019) adds a level of complexity to this scenario. In this regard, pp65 is able to inhibit NF-κB but not IRF3 nuclear translocation (Browne and Shenk, 2003). This is in disagreement with findings by Abate et al. (2004) showing that pp65 reduces IRF3 phosphorylation preventing its nuclear translocation.

Recent results obtained by our group have demonstrated that the pyrin association domain (PAD) of pp65 binds cGAS, thereby inhibiting its enzymatic activity upon HCMV infection. This phenomenon leads to impairment of the cGAS/STING axis and downregulation of IFN-β production (Biolatti et al., 2018b). In good agreement with these findings, the HCMV tegument protein pUL31 (encoded by *UL31*), similar to pp65, can interact with nuclear and cytoplasmic cGAS in HCMV-infected HFFs and HEK293T cells. Results from Huang et al. (2018) have shown how pUL31 can interact directly with cGAS in HEK293T cells, which is followed by disassociation of DNA from cGAS leading to decreased cGAMP production and consequent downregulation of IFN-I gene expression.

The HCMV tegument protein pp71 (i.e., pUL82, encoded by *UL82*) also contributes to evade the IFN response. According to Fu et al. (2017), pp71 interacts with the inactive rhomboid protein 2 (iRhom2) and STING to disrupt STING trafficking. Particularly, pp71 prevents STING translocation from the ER to the perinuclear microsomes, an essential step of STING-mediated signaling.

The HCMV glycoprotein US9, encoded by *US9*, inhibits IFN-I by targeting mitochondrial antiviral-signaling protein (MAVS) and STING pathways (Choi et al., 2018). In this regard, Choi et al. (2018) have proposed that US9 inhibits IRF3 nuclear accumulation by preventing STING dimerization. Moreover, the overexpression of US9 disrupts the mitochondrial membrane integrity and its membrane potential.

Moreover, the HCMV immediate early (IE) 86 kDa protein (IE86), negatively affects IFN-β mRNA transcription by preventing NF-κB binding to the IFN-β promoter (Taylor and Bresnahan, 2006). Intriguingly, a recent study by Kim et al. (2017) has shown that IE86 downregulates STING protein, suggesting that IE86 may also target STING for proteasomal degradation. Interestingly, STING levels were restored upon treatment with the peptide aldehyde MG132, which prevents the proteolytic activity of the proteasome complex. However, no interaction between STING and IE86 during HCMV infection could be detected.

Finally, HCMV tegument proteins have also been proposed to affect the modulation of type II IFN (also known as IFN-γ) signaling, which is an aspect not well studied. In this regard, Feng et al. (2018) have reported that the human N-myc interactor (Nmi) protein, which is important for the activation of IFN-γ, specifically interacts with the viral tegument protein UL23, encoded by *UL23*, leading to a decrease in IFN-γ expression, thus facilitating viral immune evasion.

To summarize, HCMV has evolved sophisticated mechanisms to modulate the host IFN response, especially that through IFN-I. This new evidence contributes to our understanding of the molecular mechanisms employed by HCMV to evade the innate immune response (Table 1).

**TABLE 1:** Summary of studies describing HCMV evasion strategies from IFN antiviral activity.

**Viral protein (viral gene)**	**Host target**	**Suggested mechanism**	**Type of IFN**	**References**
pp65 (UL83)	NF-κB	Reduced nuclear relocalization	IFN-β	Browne and Shenk, 2003
	IRF3	Reduced phosphorylation and relocalization	IFN-β	Abate et al., 2004
	cGAS	Reduced enzymatic activity	IFN-β	Biolatti et al., 2018a
pUL31 (UL31)	cGAS	Dissociation of cGAS from DNA	IFN-β	Huang et al., 2018
pp71 (UL82)	iRhom	Distruption of translocation complex	IFN-β	Fu et al., 2017
	STING	Distruption of translocation complex	IFN-β	
US9 (US9)	MAVS	Attenuation of MAVS signaling	IFN-β	Choi et al., 2018
	STING/TBK1	Prevention of STING oligomerization	IFN-β	
	IRF3	Dysfunctional nuclear relocalization	IFN-β	
IE86 (UL122)	NF-κB	Preventing interaction with IFN-β promoter	IFN-β	Kim et al., 2017
	STING	Proteasome degradation	IFN-β	Taylor and Bresnahan, 2006
UL23 (UL23)	Nmi	Disruption of Nmi/STAT1 interaction	IFN-γ	Feng et al., 2018

## Restriction Factors vs. HCMV: A Never Ending Fight

During the last few years, RFs have emerged as main players of the host antiviral response against HCMV (Paludan et al., 2011). RFs are intrinsic antiviral factors, which are sometimes regarded as integral part of the innate immune response or some other times an autonomous third branch of the immune system (Yan and Chen, 2012). Unlike other classical components of innate immunity, they are constitutively expressed within the host cells and are generally IFN inducible, thus allowing an immediate response against viral infection through specific targeting of viral/cellular components (Bieniasz, 2003; Hotter and Kirchhoff, 2018). Interestingly, during HCMV infection a subset of classical IFN-stimulated genes (ISGs) may be also induced or upregulated independently of IFN (Ashley et al., 2019).

**FIGURE 2:**
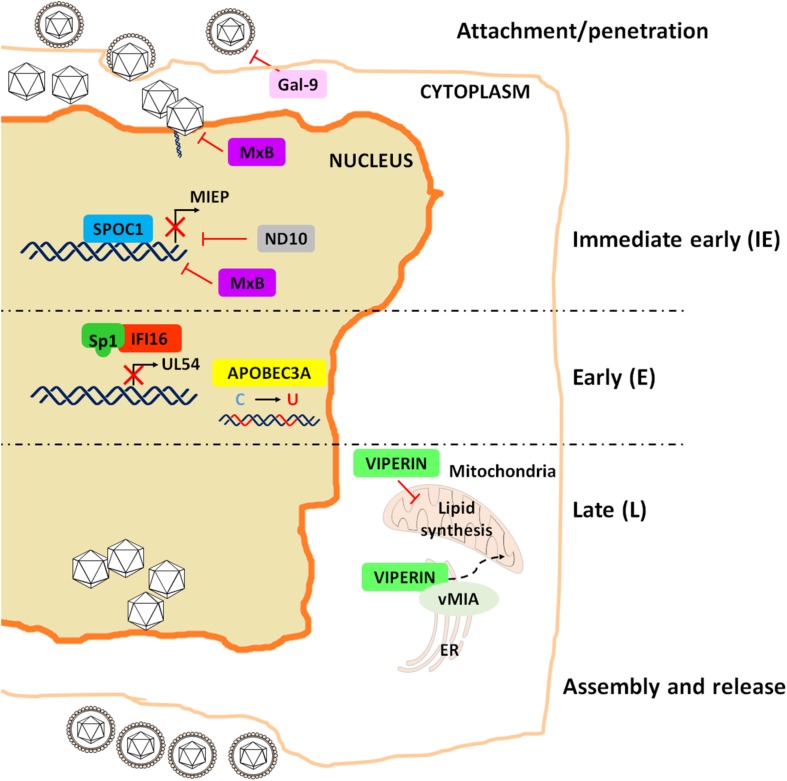
Schematic representation of the restriction activities played by the major RFs to impair HCMV replication.

Similar to what observed for the IFN system, HCMV has devised clever strategies to sidestep the antiviral activity of RFs, among which IFN-γ-inducible protein 16 (IFI16), nuclear domain 10 (ND10) and virus inhibitory protein ER-associated IFN-inducible (viperin) are among the best characterized (Biolatti et al., 2018c). This list has been in the last few years expanded to include apolipoprotein B editing catalytic subunit-like 3 (APOBEC3), survival time-associated PHD protein in ovarian cancer 1 (SPOC1), Galectin-9 (Gal-9) and human myxovirus resistance 2 (MX2) gene product MxB (Figure 2).

Unexpectedly, BST2/tetherin, considered to be the pioneer among RFs due to its long established antiviral activity against human immunodeficiency virus (HIV), does not display restriction activity against HCMV, but it rather enhances the susceptibility of hematopoietic cells to HCMV infection, thereby favoring viral hematogenous spread (Viswanathan et al., 2011). Similarly, IFN-inducible transmembrane proteins (IFITMs) 1, 2, and 3, capable of blocking the entry of a broad variety of RNA viruses, fail to inhibit the entry of DNA viruses, such as HCMV, HPV16 and human adenovirus type 5, pointing to an evolutionarily preserved mechanism shared by some DNA viruses to circumvent the antiviral function of IFITMs (Warren et al., 2014). This is however a controversial point, as a more recent study has shown that HCMV, instead of taking part in the entry process, exploits IFITMs at later time points of its viral cycle to facilitate the formation of the virion assembly compartment (vAC), which enhances virion assembly (Xie et al., 2015). Finally, a very recent work elegantly described the ability of HCMV to actively stimulate the cellular RNA-binding protein Roquin in inhibiting the innate immune response through the suppression of IRF1 antiviral activity (Song et al., 2019).

### IFI16

In the past decade, our group and others have extensively investigated the antiviral activity of IFI16 against HCMV. In particular, we have shown that IFI16 inhibits HCMV replication at early-late phases through blockade of Sp1 binding to the HCMV DNA polymerase promoter (UL54) (Gariano et al., 2012). At late stages of infection, we also found that HCMV is able to promote IFI16 nuclear delocalization through UL97-mediated IFI16 phosphorylation. Phospho-IFI16 is then redirected from the nucleus to the vAC where it is incorporated into newly formed viral particles (Dell’Oste et al., 2014).

This unexpected behavior raised the important question of why HCMV chooses to incorporate an RF (i.e., IFI16) into its virions. A partial answer to this riddle came from experiments on pp65 showing that at early stages of HCMV infection this tegument protein can interact with IFI16 at the major immediate-early promoter/enhancer (MIEP), promoting viral gene transcription. Thus, entrapping cytoplasmic IFI16 into virions might after all confer a fitness advantage to the virus (Cristea et al., 2010). However, more recent findings have shown that pp65 can also protect IFI16 from degradation, thereby favoring the inhibitory effect of this latter on the promoter region of *UL54* (Biolatti et al., 2016). Interestingly, it has been recently demonstrated that IFI16 is rapidly targeted during the establishment of viral latency in a US28-dependent manner, but only in undifferentiated myeloid cells, a natural site of latent carriage (Elder et al., 2019). These authors have indeed proposed that the consequent downregulation of IFI16 is beneficial to the establishment of latency, since IFI16 overexpression drives MIEP activity and IE gene expression via NF-κB.

In addition to its antiviral activity, IFI16 is also able to induce IFN-β expression through cGAS interaction (Diner et al., 2016). cGAS activity plays a major role in the STING/tank-binding kinase (TBK-1)/IRF3 pathway, activated by herpes simplex virus type 1 (HSV-1) and HCMV infection (Diner et al., 2016; Biolatti et al., 2018c). Therefore, it does not come as a surprise that also in this case HCMV has been able to develop a strategy to counteract cGAS activity. Indeed, HCMV UL31 has been recently identified as a cGAS inhibitor, acting through direct protein-protein interaction followed by DNA dissociation from cGAS and reduced cGAMP production (Huang et al., 2018).

### ND10 Complex

One of the best characterized HCMV RFs is certainly the ND10 complex, formed by the proteins PML, hDaxx, and Sp100 (Zhang and van Drunen Littel-van den Hurk, 2017). In addition to these components, other molecules, such as the nuclear matrix protein microrchidia family CW-type zinc-finger 3 (MORC3/NXP-2), have been shown to associate with the ND10 complex and exert antiviral activity through an unknown mechanism (Sloan et al., 2016).

During HCMV infection, the viral genome is accumulated at the periphery or within the central core of ND10 bodies, and all the ND10 components are recruited at the site of viral replication to exert their antiviral activity (Tavalai et al., 2008; Adler et al., 2011; Cosme et al., 2011; Glass and Everett, 2013). This is achieved by forming a transcriptionally inactive chromatin complex binding the MIEP, which then silences IE gene expression (Preston and Nicholl, 2006; Woodhall et al., 2006; Lukashchuk et al., 2008; Shin et al., 2012). Moreover, PML is an E3 ligase mediating IE1 SUMOylation, thereby blocking the antagonistic effect of IE1 on STAT-mediated IFN response (Reuter et al., 2017).

Although PML, hDaxx, and Sp100 act as RFs during HCMV lytic replication, they do not seem to affect HCMV latency, as demonstrated by silencing experiments in non-differentiated THP-1 monocytes (Wagenknecht et al., 2015). Meanwhile, other have shown that hDaxx can act as an RF in several latency cellular models, such as NT2 and THP-1 cells, myeloblastic cell lines and primary human CD34^+^ cells (Saffert and Kalejta, 2006).

Also in this instance, HCMV has developed fine-tuned strategies to subvert the gatekeeping functions of ND10. Perhaps the most surprising solution adopted by HCMV relies on IE1, probably because this viral protein is also the main target of the ND10 complex. Specifically, IE1 can block ND10 SUMOylation (Xu et al., 2001; Lee et al., 2004; Schilling et al., 2017), thereby preventing ND10 oligomerization and activation (Korioth et al., 1996; Ahn and Hayward, 1997; Wilkinson et al., 1998). Moreover, the viral latency-associated gene product (LUNA), encoding a deSUMOylase activity, promotes the disruption of cellular ND10 bodies during latency (Poole et al., 2018).

Other strings to the bow of HCMV are its tegument proteins. Indeed, HCMV pp71 prevents hDaxx-mediated repression of MIEP by binding this protein and stimulating its proteasome degradation, leading to disruption of the ND10-MIEP complex (Hofmann et al., 2002; Cantrell and Bresnahan, 2005). In addition, two other tegument proteins, UL35 and UL35a, have been found to cooperate in regulating pp71activity. In particular, UL35 interacts with pp71, and this interaction has two different effects: at early steps of viral replication, this complex activates IE gene transcription (Schierling et al., 2004), whereas at later stages UL35 independently remodels ND10 and co-localizes with the remodeled structures, thus facilitating pp71-mediated hDaxx disruption. Intriguingly, this activity appears to be negatively regulated by UL35a, which prevents UL35 from shaping ND10 and delivers pp71 to the cytoplasm (Salsman et al., 2011).

### Viperin

Another early identified HCMV RF is the IFN-inducible iron-sulfur (4Fe-4S) cluster-binding protein viperin, whose main antiviral activity is exerted during late phases of HCMV life cycle (Chin and Cresswell, 2001). A curious aspect of this interplay is that HCMV is not just able to inhibit viperin RF activity but it has learned how to take advantage of it in different ways. Firstly, HCMV promotes viperin translocation from the ER to the mitochondria by encoding the viral mitochondria-localized inhibitor of apoptosis (vMIA) protein. Once in the mitochondria, viperin can inhibit viral replication by modulating the host metabolism through three distinct mechanisms: (1) inhibition of fatty acid β-oxidation; (2) downregulation of ATP levels; and (3) rearrangement of the actin cytoskeleton (Seo et al., 2011). To this end, viperin transcriptionally activates several mediators of fatty acid metabolism, such as AMP-activated protein kinase (AMPK) and GLUT4 (Seo and Cresswell, 2013). This processes leads to enhanced lipid production in HCMV-infected cells, which in turn favors viral envelope formation and virion release.

### APOBEC3

Together with tetherin, cytidine deaminases belonging to the APOBEC3 family are considered fundamental antiviral proteins, known for their antiviral activity against HIV-1 (Blanco-Melo et al., 2012). Over the years, their antiviral activity has also been shown to affect DNA viruses, including HCMV (Harris and Dudley, 2015). Specifically, the APOBEC3 family member APOBEC3A (A3A) is upregulated in the maternal decidua upon HCMV infection or IFN-β administration and displays a strong inhibitory effect against HCMV replication (Weisblum et al., 2017). Furthermore, A3A cytidine deamination activity is responsible for hypermutations in the viral genome of HCMV-infected epithelial cells, thereby impairing HCMV replication through a poorly defined mechanism, presumably involving IFN-β (Weisblum et al., 2017).

The observation that A3A is not the only APOBEC3 isoform induced by HCMV comes from one of our recent studies showing that A3G is also strongly upregulated in HCMV-infected HFFs, an induction apparently mediated by IFN-β (Pautasso et al., 2018). However, the fact that the HCMV genome almost totally lacks A3G motifs (i.e., CCC) rules out the possibility that this protein is a *bona fide* HCMV RF, raising the hypothesis that host-virus coevolution might have shaped the nucleotide composition of HCMV DNA to generate viruses able to dodge A3G-mediated immune surveillance.

### SPOC1

SPOC1, also known as PHF13 (PHD finger 13), was characterized for the first time in patients with epithelial ovarian cancer (Mohrmann et al., 2005). Many cellular functions of this protein can be attributed to its ability to bind and modulate chromatin by cooperating with several heterochromatin proteins. By doing so, SPOC1 differentially regulates subsets of genes mainly involved in DNA binding and chromatin organization, cell cycle and differentiation (Kinkley et al., 2009; Bördlein et al., 2011; Chung et al., 2016). SPOC1 is also a DNA repair factor as it accumulates at DNA double-strand breaks and regulates the DNA damage response (Mund et al., 2012). A restriction activity of SPOC1 has been observed against human adenovirus type 5 (HAdV5) (Schreiner et al., 2013) and HIV-1 (Hofmann et al., 2017). In these specific contexts, SPOC1 inhibits viral replication, but it is also degraded by viral proteins as a negative feedback mechanism. Furthermore, SPOC1 inhibits early steps of HCMV replication by specifically binding MIEP and driving the recruitment of heterochromatin-building factors, in line with its chromatin remodeling activity. Intriguingly, HCMV but not HIV-1 and AdV5 infection promotes and early transient upregulation of SPOC1 through an IE1-mediated mechanisms independent of protein stabilization. At later steps of infection, SPOC1 levels start to decline upon phosphorylation by the serine-threonine kinase glycogen synthase kinase 3β (GSK-3β) (Hofmann et al., 2017). However, contrary to HIV-1 infection, where Vpr has already been identified as the viral protein involved in SPOC1 degradation (Reichel et al., 2018), the mechanism of HCMV-mediated downregulation of SPOC1 still remains obscure.

### Gal-9

Among the most recently identified HCMV-RFs, Gal-9 is of particular interest. It belongs to the widely expressed protein family of galectins, playing an important role in both innate and adaptive immunity (Rabinovich et al., 2007; Rabinovich and Toscano, 2009). The immunomodulatory role of Gal-9 is due to the presence of glycan structures on the surface of both host cells and microorganisms, thus enabling galectins to orchestrate antiviral immunity as well as host-virus interactions. For example, Gal-1 and Gal-9 have shown antiviral activity against Epstein-Barr virus (EBV), murine CMV infection (MCMV), Nipah virus (NIV), enterovirus, HIV-1, influenza virus, and dengue virus in a number of *in vivo* and *in vitro* models of infection (reviewed in Merani et al., 2015).

Even though galectins can either enhance or inhibit viral infection, a restriction activity of Gal-9 during HCMV infection has been recently observed (Machala et al., 2019). In experiments where Gal-9 was added at different time points after HCMV infection it functioned as an antiviral lectin binding the virions and blocking entry of HCMV into the host cell without influencing post-entry events (Machala et al., 2019). On the other hand, the same authors observed increased concentrations of soluble Gal-9 in the plasma of hematopoietic stem cell transplantation (HSCT) recipients during HCMV reactivation, raising the possibility that Gal-9 may also exert a restriction activity *in vivo* (Machala et al., 2019).

### MxB

The Mx GTPases MxA and MxB are best known as RFs of several RNA viruses, including influenza A virus, vesicular stomatitis virus (VSV), measles virus (MeV) (Haller and Kochs, 2011), and HIV-1 (reviewed in Staeheli and Haller, 2018). The antiviral activity of Mx against herpesviruses is somewhat more controversial. Indeed, while it has recently been demonstrated a pan-herpesvirus restriction activity for MxB against IE viral gene expression, the precise mechanisms it relies on has not yet been fully clarified (Schilling et al., 2018). The most consistent hypothesis is that of a direct action of MxB during the uncoating process aimed at targeting viral capsids or components of the nuclear pore complexes, similarly to what happens during HSV-1 infection (Crameri et al., 2018).

## Antigen Presenting Cells: a Two-Edged Sword

APCs are often defined as sentinels of the body, essential for initiating the immune response against pathogens. They, however, play an enigmatic role during HCMV infection. On the one hand, many APCs, including monocytes, macrophages and DCs, are critical to trigger specific T-cell responses. On the other hand, they are permissive to HCMV infection, serving as vehicles for viral spread during the first steps of infection, and then becoming cozy and protective niches for virus replication and persistence at later stages. Conversely, components of the lymphoid lineage, such as NK cells and plasmacytoid DCs (pDCs) are not just resistant to HCMV infection but they are also activated early upon infection by viral components, triggering an antiviral response. Despite the presence of these defense mechanisms, HCMV has put in place multiple strategies to evade APC-mediated immune control so as to establish latency and persistence within the host (Sinclair and Reeves, 2014).

### Dendritic Cells (DCs)

DCs are specialized APCs mediating immune response induction and maintenance. The major subsets in humans include classical DCs (cDCs), which comprise Langerhans cells (LCs) and pDCs, the main producers of IFN-I, and monocyte-related DCs (mDCs) (Collin et al., 2013). The role of DCs during HCMV infection remains somewhat controversial because, despite being critical components for the establishment of an antiviral NK and T-cell response, they are also targeted by HCMV for immune escape.

HCMV interacts with DCs in a pleiotropic manner. It is in fact well established that HCMV strains with an intact UL128-131A locus can infect DCs *in vitro* (Jahn et al., 1999; Riegler et al., 2000). In addition, circulating mDCs isolated from healthy seropositive donors can also support HCMV infection (Reeves and Sinclair, 2013), a process probably favored by the expression of the viral chemokine receptor-like protein US28, which drives DC recirculation (Farrell et al., 2018). In contrast, by using co-culture approaches, it has been shown that mDCs or monocyte-derived macrophages can restrict HCMV with interferon-unrelated mechanisms (Kasmapour et al., 2017; Becker et al., 2018).

For pDCs, the scenario is even more complex. Different subpopulations of pDCs obtained either from tonsils (tpDCs) or blood (bpDCs) react to HCMV-infection in opposite ways (Schneider et al., 2008). For instance, tpDCs are fully permissive for HCMV replication despite the fact that their IFN-α production and expression of costimulatory and adhesion molecules are ultimately affected by HCMV. In contrast, bpDCs appear to be resistant to HCMV infection (Schneider et al., 2008).

HCMV can latently infect DC precursors and then undergo reactivation by taking advantage of chromatin remodeling during differentiation of DC progenitors into mature DCs (Reeves et al., 2005). Conversely, in undifferentiated myeloid precursors, viral lytic genes are inhibited as a consequence of histone modifications of the MIEP, leading to a repressive chromatin structure eventually preventing IE transcriptional activity (Sinclair, 2010). Furthermore, proinflammatory factors, such as IL-6 and the ERK/MAPK pathway have been linked to the reactivation of latent HCMV in DCs and other permissive cells (Reeves and Compton, 2011).

The interplay between HCMV and DCs interaction can have different outcomes in terms of immune response. For instance, HCMV infection of mDCs *in vitro* triggers IFN and IL-12 release in a cGAS-dependent manner (Renneson et al., 2009; Paijo et al., 2016). Subsequently, other immune mediators are recruited to the infection site to amplify the immune reaction. HCMV infection in mDCs can also modulate TLR3 signaling, but this effect is more evident at later times post-infection (Mezger et al., 2009).

Given the central role of DCs in virus clearance, it is not surprising that HCMV has put in place multiple strategies to inhibit such process. For instance, HCMV can interfere with MHC-I and -II antigen processing and presentation to avoid detection by CD8^+^ and CD4^+^ T cells. This process appears to be mediated by the HCMV-encoded protein US2, capable of degrading both MHC-I and MHC-II proteins through the proteasome (Loureiro and Ploegh, 2006). Likewise, other HCMV proteins such as pp65, pp71, and US2-11 have been implicated in HCMV evasion from T-cell recognition by triggering accumulation and degradation of HLA-DR α-chain in perinuclear vacuoles (Odeberg et al., 2003).

Among HCMV genes hindering APC function, a crucial role is played by the viral interleukin-10 homolog (cmvIL-10), expressed during lytic infection and capable of binding the IL-10 receptor of host cells. Specifically, cmvIL-10 upregulates the HCMV putative receptor DC-SIGN, thus enhancing viral infectivity (Raftery et al., 2004), as well as the expression of hIL-10 by primary blood-derived monocytes, thus modulating existing cellular pathways and the viral immunomodulatory impact during infection (Avdic et al., 2016). In addition, it inhibits a number of DCs functions, including TLR-induced IFN-α/β production in nearby pDCs and CD1-mediated antigen presentation (Raftery et al., 2008; Avdic et al., 2014). This effect is also shared by other viruses, which either upregulate hIL-10 (e.g., HIV and hepatitis C virus) (Reiser et al., 1997; Brockman et al., 2009) or express homologs of this cytokine (e.g., EBV and some cytomegaloviruses) (Slobedman et al., 2009), highlighting the importance of IL-10 signaling in viral escape mechanisms.

**FIGURE 3:**
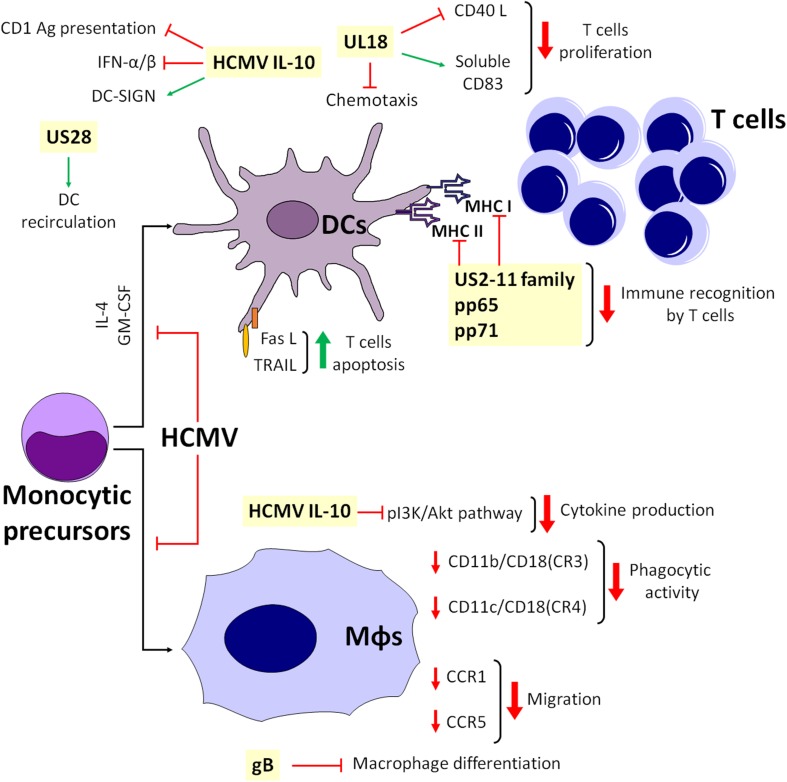
Simplified model depicting the interplay among APCs during HCMV infection.

An important step of the immune response is the ability of DCs to drift from the infection site to the lymph nodes, a process driven by the chemokines CCL19 and CCL21. Consequently, HCMV has developed strategies to impede DC trafficking in response to lymphoid stimuli and induction of T-cell proliferation (Beck et al., 2003; Moutaftsi et al., 2004). For example, it can prevent CCR5 chemokine receptor from switching to CCR7 in infected mDCs, thus inhibiting CCL19- and CCL21-induced migration of mature mDCs (Moutaftsi et al., 2004). Conversely, in immature mDCs, HCMV does not modulate CCR7, but it affects chemotaxis by internalizing CCR1 and CCR5 (Varani et al., 2005). In this context, UL18, the viral homolog of MHC-I, appears to play a controversial role. Indeed, UL18 has been reported to inhibit CD40L-mediated T-cell proliferation through DC maturation impairment (Wagner et al., 2008), meanwhile stimulating the expression of CD83 on mature mDCs. Moreover, at later times, HCMV downregulates surface but not intracellular CD83 (Wagner et al., 2008). Others have reported that soluble CD83, in turn, inhibits T-cell proliferation (Sénéchal et al., 2004), and that UL18 is also able to reduce RANTES-driven chemotaxis of mDCs (Wagner et al., 2008; Figure 3).

Depending on their stage of maturation, CD34^+^ progenitor cell-derived LCs can be susceptible to HCMV infection. Indeed, immature LCs are poorly supportive of viral replication, whereas LC-derived mature DCs are highly responsive to infection due to HCMV-mediated subversion of the T-cell response through downregulation of several activation markers, such as MHC-I and -II, CD1a, CD80, CD83, CD86, and CD54 (Hertel et al., 2003). This also leads to a substantial loss of dendrites and to impaired dendritic cell migration in response to lymphoid chemokines (Lee et al., 2004; Figure 3).

### Monocytes and Macrophages

Additional reservoirs for HCMV are represented by monocytes and macrophages. In particular, monocytes have been long involved in HCMV dissemination across the human body and are generally regarded as the main source of latent HCMV in the peripheral blood of seropositive people (Smith et al., 2004). Even though they do not support productive HCMV replication (Sinzger et al., 2008), once fully differentiated into macrophages, they become permissive for viral replication. During this process, a major role for virus reactivation and growth seems to be played by IFN-γ and tumor necrosis factor (TNF)-α, produced by allostimulated T cells (Söderberg-Nauclér et al., 1997). Moreover, monocytes are known to release infectious HCMV directed toward uninfected cells *in vitro* through a not fully defined mechanism (Waldman et al., 1995).

Like DCs, monocyte-derived macrophages play a crucial role in counteracting HCMV spread *in vitro*. In this context, the role of IFN is controversial. Indeed, IFN-I plays an inhibitory role on HCMV replication when macrophages are stimulated by cell-free HCMV. In contrast, upon co-culture of infected cells and macrophages, the antiviral effect appeared to be independent of IFN-γ, TNF-α, and IFN-I (Becker et al., 2018).

Overall, it seems that HCMV has learned how to escape from monocyte antiviral activity and use these cells as “Trojan horses” to achieve viral spread. For instance, infected monocytes display impaired migration and reduced ability to recruit leukocytes and inflammatory mediators, allowing additional “contact time” to transfer HCMV from infected monocytes to uninfected cells (Frascaroli et al., 2006). Furthermore, the observation that purified pUL128 – i.e., a CC chemokine homolog, part of the HCMV pentamer complex (PC) – triggers monocyte migration *in vitro* through a poorly characterized mechanism suggests that HCMV might be able to attract monocytes to the infection site and favor viral dissemination by secreting specific chemokines (Zheng et al., 2012). In addition, pUS2-US11-mediated MHC downregulation in DCs is only partially functional in macrophages, which therefore retain their ability to activate CD4^+^ and CD8^+^ T cells (Frascaroli et al., 2018). Lastly, HCMV inhibits the differentiation of both macrophages and DCs from monocytic precursors after stimulation with IL-4 and GM-CSF, impairing immunological functions (Gredmark and Söderberg-Nauclér, 2003). In this context, the main inhibitors of macrophage differentiation are the cell-surface aminopeptidase N/CD13 and HCMV glycoprotein B (gB) (Gredmark et al., 2004; Figure 3).

As for DCs, cmvIL-10 can also impair cytokine production of these cells through inhibition of phosphatidylinositol 3-kinase/Akt signaling (Spencer, 2007), with concurrent downmodulation of integrin-like receptor surface expression [i.e., CD11b/CD18 (CR3) and CD11c/CD18 (CR4)], a process that strongly impairs DC phagocytic activity (Gafa et al., 2005). Finally, downregulation of CCR1 and CCR5 is associated with slower cell migration, reorganization of the cytoskeleton and secretion of soluble inhibitors (Frascaroli et al., 2009; Figure 3).

## Nk Cells and Hcmv: a Balance of Opposing Forces

NK cells play crucial role in eliminating HCMV-infected cells through cytotoxicity and secretion of several cytokines and chemokines able to directly impair viral replication (e.g., IFN-g and TNF-a) or to recruit and/or activate other cells of the immune system. However, if on one side there are examples demonstrating the importance of NK cells in controlling HCMV infection, on the other side there is a long list of viral proteins capable of protecting HCMV from NK cell recognition and killing (Brown and Scalzo, 2008; Schmiedel and Mandelboim, 2017; Patel et al., 2018).

The former case is best exemplified by human NK cell primary immunodeficiencies (NKD), which inevitably results in high susceptibility to herpesvirus infections [i.e., HCMV, HSV, EBV, and varicella zoster virus (VZV)] (Biron et al., 1989). In this regard, more than 60% of NKD patients are infected by one of these viruses (Orange, 2013), also in the context of intact CTL functions (Quinnan et al., 1982). The severity of this condition is demonstrated by the fact that nearly half of patients with NKD tend to die prematurely (Orange, 2013; Mace and Orange, 2019).

The antiviral activity of NK cells against HCMV also appears to be mediated by NK cell receptors, whose expression can be to some extent modulated upon viral entry. In particular, HCMV infection can induce the selective expansion of a population of NK cells expressing the activating receptor CD94/NKG2C, giving rise to the so-called “adaptive-like” or “memory-like” NK cells (Gumá et al., 2004). This aspect of NK and HCMV biology is beyond the scope of this review and has already been extensively described in recent reviews (López-Botet et al., 2014, p. 94; O’Sullivan et al., 2015; Rölle and Brodin, 2016).

What is important to point out in this context is that NKG2C receptor skewing is accompanied by other phenotypic, functional and epigenetic modifications, which lead to the generation of a pool of long-living NK cells with increased effector responses upon restimulation. Importantly, Hammer et al. (2018) have recently shown that the triggering event driving NKG2C^+^ NK cell expansion is mediated by an HCMV-encoded peptide derived from the viral protein UL40 and by the NKG2C ligand HLA-E. However, it is worth pointing out that the emergence of NK cell memory in response to HCMV can also occur in individuals lacking expression of NKG2C – i.e., carrying the null allele KLRC2 encoding for NKG2C – (Noyola et al., 2012), suggesting that alternative or compensatory mechanisms may be in place. This mode of activation is nonetheless complex, as HLA-E is also recognized by CD94/NKG2A, the inhibitory counterpart of CD94/NKG2C, with identical peptide specificity (Braud et al., 1998; Lee et al., 1998; Brooks et al., 1999; Cerboni et al., 2000; Ulbrecht et al., 2000; Tomasec et al., 2005). Stabilization of HLA-E by the UL40-derived peptide can thus have opposite effects on NK cells, depending on which receptor is involved. However, it seems that the NKG2C^+^ NK cell population expanding in HCMV seropositive individuals lacks the inhibitory NKG2A heterodimer (Hammer et al., 2018). In addition, the peptide repertoire encoded by different HCMV UL40 variants may result in an intermediate state, where peptides able to efficiently inhibit NKG2A and simultaneously trigger suboptimal activation of NKG2C^+^ NK cells are more prevalent (Hammer et al., 2018).

The important role of NK cells in CMV infection comes also from a plethora of studies conducted in mice. In general, the absence of NK cells – due to genetic or neutralizing/depleting antibody manipulations – results in a significantly diminished, and sometimes lethal, control of MCMV (Bukowski et al., 1984; Brown and Scalzo, 2008). Similarly to HCMV, it has been reported a pathogen-specific recognition mechanism for protection, involving the NK cell-activating Ly49H receptor, which specifically recognizes the MCMV protein m157 (Arase et al., 2002).

**FIGURE 4:**
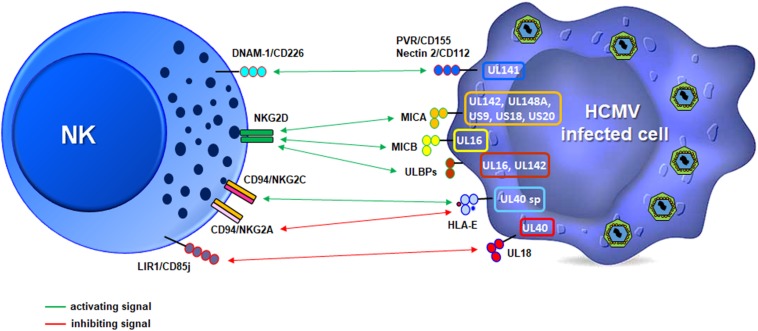
Schematic illustration of the strategies used by HCMV to modulate NK cell receptor ligands. The viral proteins mainly involved are depicted in a representative infected cell (right, color outlines), and activating (green) or inhibitory (red) signals relative to specific receptors on a NK cell (left) are shown.

Another important strategy for immune escape is the ability of HCMV to manipulate the expression of several ligands of the NKG2D receptor, expressed on all NK cells, CD8^+^ T cells and other T-lymphocyte subsets (e.g., CD4 + T cells, gd, and NKT cells) (Lanier, 2015; Zingoni et al., 2018). There are eight different NKG2D ligands (i.e., MICA, MICB, and ULBP1-6), all belonging to the MHC class I-like family and possessing two or three αdomains, but not able to bind peptides or β2-microglobulin. These molecules are also known as “stress-induced ligands” or “induced self” as they are rarely expressed on the plasma membrane of healthy cells but can be rapidly up-regulated upon different types of stress, including those triggered by viral infection (Cerboni et al., 2014; Lanier, 2015). In the absence of a specific viral countermeasure, up-regulation of NKG2D ligands (NKG2DLs) would likely result in the killing of infected cells, as it has been observed in some experimental conditions (Cerboni et al., 2000; Wang et al., 2002; Pignoloni et al., 2016). However, *in vitro* studies have shown that this is not always the case since HCMV encodes at least seven different molecules – among which a few identified very recently – able to inhibit NKG2DL expression, thus conferring protection to the infected cells. In particular, MICA seems to be the most frequently targeted ligand, with UL142, UL148a, US9, US18, and US20 viral proteins dedicated to block its expression at different levels, sometimes in an allelic-specific manner (Schmiedel and Mandelboim, 2017; Patel et al., 2018; Figure 4). Although the reason for such a high number of HCMV proteins targeting just one ligand is currently unknown, their existence may be ascribed to the fact that, among NKG2D ligands, MICA has the highest affinity for its receptor (Steinle et al., 2001) as well as the largest number of variant alleles, with more than 100 identified thus far^1^. Based on these findings, it is tempting to speculate that the antiviral activity of MICA may have selected viruses able to block MICA expression and the ensuing NKG2D-mediated killing, and that this in turn might have promoted MICA polymorphism.

Among NKG2D ligands, we find MICB, a polymorphic gene with more than 40 allelic variants, and 6 ULBP genes boasting a total of 16 allelic variants^2^ (Radosavljevic et al., 2002). MICB expression is inhibited by miR-UL112, the only HCMV-encoded miRNA described to date targeting this ligand (Stern-Ginossar et al., 2012), and by the viral protein UL16, which is a sort of promiscuous immunoevasin since it can also inhibit the expression of ULBP1, ULBP2, and ULBP6 (Cosman et al., 2001; Kubin et al., 2001; Dunn et al., 2003; Rölle et al., 2003; Wu et al., 2003; Eagle et al., 2009). ULBP3 is instead targeted by UL142, also blocking MICA expression (Ashiru et al., 2009; Bennett et al., 2010). The ability to simultaneously evade multiple cellular pathways has also been reported for US18 and US20, capable of inhibiting both MICA and the NKp30 ligand B7-H6 (Charpak-Amikam et al., 2017; Fielding et al., 2017).

Other targets of HCMV include CD155/PVR and CD112/Nectin-2, two adhesion molecules belonging to the Ig-like superfamily able to bind the activating receptor CD226/DNAM-1 expressed on cytotoxic lymphocytes (Figure 4; Shibuya et al., 1996; Bottino et al., 2003; Tahara-Hanaoka et al., 2004). Similar to NKG2DLs, DNAM-1 ligands (DNAM-1Ls) are often induced by cellular stresses and can trigger cytotoxicity and cytokine release (Shibuya et al., 1996; Bottino et al., 2003; Iguchi-Manaka et al., 2008). For this reason, DNAM-1Ls are also targeted by HCMV, with UL141 downregulating both of them, alone or in combination with US2 through different mechanisms (Tomasec et al., 2005; Prod’homme et al., 2007; Hsu et al., 2015). Of note, UL141 is also able to downregulate the TRAIL receptors R1 and R2, thus preventing TRAIL-dependent NK-cell killing (Nemčovičová et al., 2013; Smith et al., 2013). UL141 is thus a remarkable immunoevasion protein as it targets at least four different molecules regulating NK cell-mediated cytotoxicity.

Adhesion molecules involved in the formation of NK-target cell conjugates are also affected by HCMV. In particular, UL148 down-regulates CD58/LFA-3, the ligand of the CD2 receptor expressed by different leukocyte populations, including NK and CD8^+^ T cells. The CD2/CD58 axis promotes cell-to-cell adhesion and immunological synapse formation, providing an important co-stimulatory signal on effectors (Siliciano et al., 1985; Selvaraj et al., 1987; Browne et al., 1990) (Leitner et al., 2015). More recently, CD2 has been shown to play a role in costimulation of adaptive NK cells (Rölle et al., 2003; Liu et al., 2016). Furthermore, inhibition of CD58/LFA-3 expression by the viral protein UL148 has revealed that the CD2/CD58 axis is also needed for the recognition of HCMV-infected cells by NK cells and HCMV-specific CTLs (Wang et al., 2018).

In summary, it appears that there is a steadily increasing number of HCMV-encoded proteins evading NK cell recognition and killing. However, to date there is no single viral protein or RNA able to interfere with all the molecules involved in the anti-viral NK cell response.

It is also important to point out that development, proliferation and effector functions of NK cells are tightly regulated by both activating and inhibitory receptors, with an outcome that strongly depends on the balance between opposing signals. Inhibition is delivered *via* MHC-I molecules expressed on the surface of target cells. However, HCMV, like many other viruses, negatively affects MHC-I expression in infected cells, as this is a crucial step to avoid cell-mediated killing by viral-specific cytotoxic T cells. In theory, this would render infected cells more susceptible to NK cell recognition due to the absence of inhibitory signals. However, the observation that HCMV-infected cells are resistant to NK lysis *in vitro* seems to suggest otherwise (Cerboni et al., 2000; Wang et al., 2002). What we have in fact described in this section is a plethora of viral molecules evolved by HCMV to escape from NK cell activation, which otherwise would be detrimental for viral fitness.

To complete this picture, HCMV can fully accomplish immunoevasion from NK cells thanks to its own MHC-I surrogate, called UL18. This protein is markedly similar to cellular MHC-I molecules (Beck and Barrell, 1988; Browne et al., 1990) and acts as a viral homolog by binding with high affinity the MHC-I NK cell inhibitory receptor CD85j/LIR1/ILT2, thereby suppressing NK cell functions (Chapman et al., 1999; Cosman et al., 2001; Cerboni et al., 2006; Prod’homme et al., 2007).

In conclusion, HCMV is a driving force in shaping the NK cell receptor repertoire and modes of recognition of infected cells. The virus is not only capable of “hitting the brakes” of NK cells through its own MHC-I surrogate (UL18) or by engaging the CD94/NKG2A inhibitory receptor with UL40, but it can also “block the gas pedal” by inhibiting the expression of several ligands of NK cell activating receptors. The outcome is a million-year-long host-pathogen equilibrium, where neither the host nor the pathogen is at risk of extinction.

## HCMV and Apoptosis: “Not Today!”

Apoptosis, or programmed cell death (PCD), is essential for the maintenance of homeostasis and survival of most multi-cellular organisms. Apoptosis occurs predominantly through the following three pathways: (1) extracellular ligand-mediated extrinsic pathway; (2) mitochondria-mediated intrinsic pathway; and (3) ER-mediated pathway. The extrinsic pathway is initiated upon binding of extracellular ligands to death receptors (DRs), leading to the formation of the death-inducing signaling complex (DISC), required for the activation of initiator caspases (i.e., cysteine proteases), caspase-8 and caspase-10. The intrinsic pathway is regulated by B-cell lymphoma 2 (Bcl-2) proteins and is characterized by mitochondrial outer membrane permeabilization (MOMP) (Elmore, 2007). The ER-mediated pathway is instead induced by stress signals, such as excessive unfolded proteins in the ER and triggers the activation of caspases-7, -9, and -12 (Bhat et al., 2017). All these pathways lead to the activation of the executioner caspases-3 and -7 that contribute to the majority of events taking place during apoptosis (Elmore, 2007).

**FIGURE 5:**
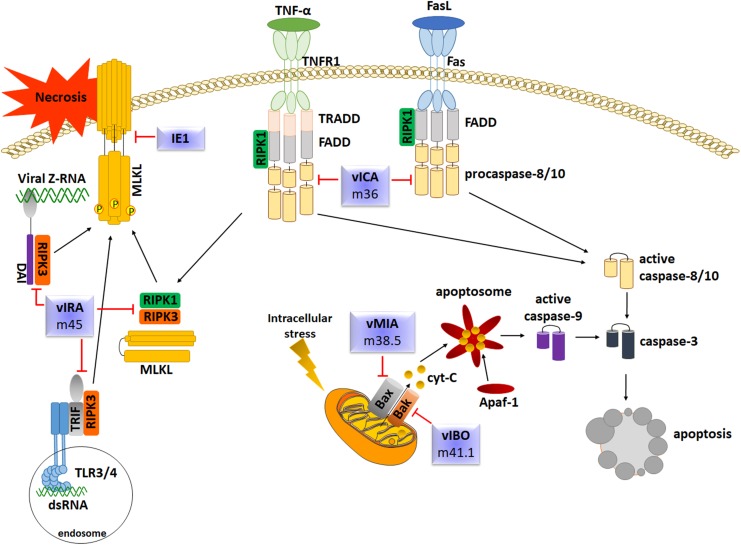
Overview of the main apoptotic pathways and evasion strategies employed by HCMV.

Apoptosis is also one of the main steps of the innate response against viral infections, including HCMV. Also in this case, HCMV has evolved several strategies to subvert host cell apoptotic defenses by targeting key effector molecules in the apoptotic cascade. Upon infection, the slowly replicating HCMV modulates cellular apoptosis pathways in various cell types, such as endothelial cells, fibroblasts and macrophages by encoding numerous death inhibitors to block premature death of host cells, thus favoring its replication (Brune and Andoniou, 2017; Collins-McMillen et al., 2018; Figure 5). The following paragraphs will contain a comprehensive review and discussion of some of the main mechanisms used by HCMV to modulate or prevent the apoptotic pathways of infected host cells.

### Inhibition of Extrinsic Apoptosis

Caspase-8 is required for initiation of apoptosis in response to death factors such as Fas-L or TNF-a. Within the Fas-FADD-Caspase-8 complex, also known as DISC, caspase-8 undergoes self-cleavage to convert to the active form. Fully-cleaved caspase-8 is released from DISC to the cytosol to trigger the apoptotic signal to downstream caspase effectors or to cleave the Bcl-2-interacting protein (Bid), which leads to the release of cytochrome c from mitochondria, inducing activation of caspase-9 in a complex with dATP and Apaf-1 (Kruidering and Evan, 2000). To counteract DR- mediated apoptosis and gain a survival advantage, HCMV encodes the viral inhibitor of caspase-8-induced apoptosis vICA/pUL36, which binds the prodomain of procaspase-8, impedes the recruitment of FADD, and prevents the formation of a functional DISC. The fact that homologs of HCMV vICA have been identified in the vast majority of mammalian betaherpesviruses implies that the function of vICA is important and conserved. This is exemplified by M36, the vICA counterpart of MCMV, which also displays an anti-apoptotic activity by interacting with procaspase-8, and that has been shown to be rescued by vICA in order to allow viral replication, confirming the reliability of the murine model (Chaudhry et al., 2017).

Moreover, the replication of UL36-deficient virus can be restored by treatment with the pan-caspase inhibitor z-VAD(OMe)-fluoromethyl ketone (fmk) only in immature but not mature macrophages, suggesting that apoptosis impairs the replication of UL36-deficient virus in defined cell types. However, according to McCormick et al. (2010), it seems that cell death pathways activated by HCMV infection are altered as monocytes differentiate to macrophages. Indeed, early during differentiation, UL36-deficient virus-induced apoptosis is dependent on caspases and can be blocked by z-VAD-fmk, while at later stages of differentiation it appears to be caspase-independent.

### Inhibition of Intrinsic Apoptosis

Mitochondria play a pivotal role in the intrinsic apoptosis pathway. Initiation and execution of this pathway is regulated by the Bcl-2 effector proteins Bax (Bcl-2-associated X protein) and Bcl-2 antagonist or killer (Bak) that control MOMP. MOMP prompts the release of proapoptotic intermembrane space (IMS) proteins that promote the formation of the apoptosome – composed by cytochrome c and Apaf-1 – and activation of caspase-9. Once active, caspase-9 can directly cleave the effector caspases 3 and 7 (Estaquier et al., 2012). HCMV prevents MOMP by encoding the viral mitochondria-localized inhibitor of apoptosis (pUL37x1/vMIA). UL37x1, highly conserved among HCMV strains, is located in a complex transcription unit encoding several transcription variants expressed during the IE phase. Two functionally longer splice variants (i.e., gpUL37 and gp37M) share with pUL37x1 an NH2-terminal 162 aa sequence responsible for inhibiting apoptosis, localize partially to mitochondria and have similar, albeit weaker, anti-apoptotic activities (Goldmacher et al., 1999; Colberg-Poley et al., 2000; Reboredo et al., 2004; Kaarbø et al., 2011). pUL37x1 blocks mitochondria-mediated apoptosis by interacting at the level of the mitochondrial outer membrane (MOM) with Bax, thus preventing cytochrome-c release. It still remains to be clarified whether vMIA can inhibit Bak during infection (Sharon-Friling et al., 2006; Sharon-Friling and Shenk, 2014).

Moreover, by using U251 glioma cells a mechanisms of viral apoptosis inhibition and enhancement of cell proliferation has been shown, relying on the activity of the immediate-early protein IE86 on heterogeneous ribonucleoprotein A2/B1 (hnRNP A2/B1) and consequent alternative splicing of Bcl-x (Zhao et al., 2019).

In addition to the aforementioned strategies, HCMV is also involved in preserving the mitochondrial membrane potential and metabolism to prevent cell death. This is achieved thanks to the production of the long non-coding RNA-lncRNA Beta2.7 that enhances cell survival through interaction with gene associated with retinoid/interferon-induced mortality 19 (GRIM19). This interaction causes the stabilization of mitochondrial membrane functions, thereby preserving ATP production and conserving metabolic activity during stress conditions (Poole et al., 2016).

### Inhibition of Necroptosis

Necroptosis is an alternative form of programmed cells death that, despite mimicking features of apoptosis, cannot be prevented by caspase inhibitors. Necroptosis can be triggered following activation of DRs as well as after stimulation with LPS, poly(I:C) or CpG DNA, which are ligands of the pattern recognition receptors (PRRs) TLR3, TLR4, and TLR9, respectively. Many downstream signaling pathways cooperate with a complex formed by the receptor interacting protein kinase 1 (RIPK1), RIPK3 and mixed lineage kinase domain-like (MLKL). Necroptosis and apoptosis are strictly interconnected, as confirmed by the observation that the inhibition of caspase-8, the main mediator of the extrinsic apoptotic pathway, promotes the shift from DR-mediated cells death to necroptosis due to activation of RIPK3 and, consequently, MLKL. Phosphorylation of MLKL generates structural changes allowing its insertion into the inner leaflet of the plasma membrane leading to the disruption of cellular membranes (Green, 2019).

### Inhibition of Cellular Stress Response

Disturbances of the normal functions of the ER, causing accumulation of unfolded proteins, trigger an evolutionarily conserved cell stress response, known as unfolded protein response (UPR), which, initially aimed to damage compensation, can eventually lead to cell death to avoid viral spread. HCMV prevents this process, in part, *via* UL38, a multifunctional protein well conserved among different CMV species. In particular, viral DNA replication is severely impaired in viruses lacking UL38 (i.e., AD*dl*UL38), a feature associated with enhanced death of infected cells (Terhune et al., 2007). Moreover, pUL38 itself can inhibit cell death induced by thapsigargin, which perturbs calcium homeostasis followed by ER-mediated cell death, or by a mutant adenovirus lacking the antiapoptotic E1B-19K protein. Of note, pUL38 cannot counteract cell death triggered by anti-Fas antibodies (Xuan et al., 2009).

Overall, the aforementioned findings suggest that pUL38 hampers both intrinsic and ER-mediated cell death, but it only slightly affects extrinsic apoptosis. UL38, expressed both at early and late stages of infection, is localized in a complex transcription unit that also retains the unspliced transcripts of UL36 and several variants of UL37, expressed during the IE phase. Probably, pUL36, pUL37x1 and pUL38 act synergically to inhibit cell death at different times during infection. As described above, while pUL36 inhibits caspase-8 activation, pUL37x1 blocks mitochondria-mediated intrinsic apoptosis. Furthermore, UL38 inhibits c-Jun N-terminal kinase (JNK) signaling through interaction with the activating transcription factor 4 (ATF4), which leads to caspase-12 or caspase-2 activation (Xuan et al., 2009).

More recently, Luganini et al. (2018) have shown that HCMV encodes for a viral-Ca^2+^-permeable channel, pUS21, able to reduce Ca^2+^ content of intracellular stores and to protect cells from apoptosis. Among the US12 gene family members, which includes a set of 10 contiguous tandemly arranged genes (US12-21), pUS21 shows the highest level of identity with two cellular transmembrane BAX inhibitor motif-containing (TMBIM) proteins: Bax inhibitor-1 and Golgi anti-apoptotic protein, both involved in the regulation of cellular Ca^2+^ homeostasis and adaptive cell responses to stress conditions. Thus, alongside pUL36, pUL37x1 and pUL38, pUS21 contributes to maintaining the viability of the host cell until the virus has completed the infection cycle.

A second mechanism used by CMV to counteract ER stress response involves the downregulation of inositol-requiring enzyme 1 (IRE1) protein levels, an ER stress sensor and cell death executor (Maly and Papa, 2014). Misfolded proteins activate IRE1, which in turn oligomerizes and self-activates its RNase activity, leading to degradation of unfolded proteins and upregulation of ER chaperon to enhance protein folding. IRE1 activation also leads to the recruitment of the TNF receptor associated factor (TRAF)-2 and activation of caspase-12 or JNK. Activated JNK induces cells death by activating proapoptotic BH3 proteins while inhibiting the antiapoptotic Bcl-2. Lastly, both MCMV and HCMV homologs M50 and UL50 enhance IRE1 degradation at later times post-infection, thus preventing all IRE1 signaling events (Stahl et al., 2013).

A second form of stress response induced by HCMV infection is that elicited by DNA damage. To ensure faithful duplication and inheritance of genetic material, cells have evolved mechanisms – collectively termed the DNA-damage response (DDR) – of DNA damage detection to induce DNA repair or, if the damage is too severe, to induce cell death (Xiaofei and Kowalik, 2014). After cell entry, HCMV capsids travel to the nucleus where the linear genome is released and circularized to serve as a template for transcription and replication by a rolling circle mechanism. This process generates multiple exposed ends that can be recognized as dsDNA by activating ataxia-telangiectasia mutated protein (ATM) and rad-3 related kinases (ATR), which initiate the DNA damage signal transduction pathway by targeting proteins involved in the checkpoint response, such as checkpoint kinase 2 (Chk2). In this regard, recent studies have revealed that HCMV can neutralize host DDR at the level of Chk2. In particular, ATM and ChK2 are mislocalized from the nucleus to the cytoplasm where they colocalize with virion structural proteins, which prevents them from initiating the DNA repair process (Gaspar and Shenk, 2006; Luo et al., 2007).

## Conclusion

Here, we have provided a comprehensive overview of the main characteristics of HCMV that have allowed this virus to evolve multiple immune evasion strategies and achieve latency and seroprevalence. These include the advanced organization and large size of its genome, restricted host specificity, viral latency and sporadic reactivation.

We have also highlighted how the host innate immune response reacts against HCMV infection through different effector cells (e.g., APCs, NK cells, and phagocytes), anti-inflammatory cytokines and IFNs. Briefly, while APCs mediate early immune activation by triggering specific T-cell responses, and cytotoxic NK cells are potent eliminators of HCMV-infected cells, early release of IFN-I and other pro-inflammatory cytokines limit the infection spread through the establishment of the so-called “antiviral state.” In addition, several IFN-inducible RFs, which belong to an additional autonomous branch of innate immunity, play a central role in inhibiting viral replication. Lastly, a significant part of the innate immune response is represented by programmed cell death, as apoptotic control greatly contributes to the removal of original population of HCMV-infected cells. Thus, thanks to the presence of multiple innate immune protective mechanisms the host, in most cases, is able to counteract HCMV spread.

However, in the course of host-virus coevolution, as described in this review, HCMV has acquired an extremely wide range of counter-defense mechanisms and manipulation strategies directed against each arm of innate immunity. For instance, HCMV is able to inhibit NK cell activation by encoding numerous proteins targeting multiple host ligands, which are likely to promote viral persistence *in vivo*. The virus is also capable of subverting the immune functions of APCs by reprogramming them as efficient means of viral dissemination, while offsetting their immune surveillance by interfering with MHC-I and MHC-II antigen presentation. Moreover, HCMV can block premature death of infected cells, thereby promoting viral replication. Major interfering with IFN-signaling pathways is also accomplished *via* a wide range of viral proteins that counteract and manipulate IFN production by the host. Thus, there is growing evidence of a highly dynamic and complex interplay between the virus and the IFN system.

From all these data, it is clear that HCMV disease progression depends on the balance between antiviral immune response and viral attempts to manipulate such response to its own advantage. Given the clinical burden of HCMV in immunocompromised patients and congenitally infected infants, there is undoubtedly an urgent and unmet medical need for an effective vaccine against this virus. Significant efforts should also be directed toward the development of more effective therapeutic agents with fewer side effects capable of targeting the virus during both its lytic and latent phases. In this regard, an in-depth analysis of the interplay among HCMV, RFs and INFs resulting in immune evasion should provide potential novel druggable targets.

## Author Contributions

VD and MD developed the ideas and drafted the manuscript. VD, MB, FG, GGa, and CC wrote sections of the manuscript. GGr, AZ, and SP drew the figures. GGa and MD professionally edited the manuscript. All authors contributed to manuscript revision, read, and approved the submitted version.

## Conflict of Interest

The authors declare that the research was conducted in the absence of any commercial or financial relationships that could be construed as a potential conflict of interest.

## Footnotes

1 http://www.ebi.ac.uk/imgt/hla/html

2 https://www.ebi.ac.uk/ipd/imgt/hla/
